# Inside general practice ethics: guidelines ‘and’ ‘of’ or ‘for’ good clinical practice

**DOI:** 10.1080/17571472.2018.1437028

**Published:** 2018-02-20

**Authors:** Andrew Papanikitas, Carey Lunan

**Affiliations:** a Nuffield Department of Primary Care Health Sciences, University of Oxford, Oxford, UK; b Craigmillar Medical Group Edinburgh, Edinburgh, UK

**Keywords:** Ethics, clinical guidelines, medicolegal, policy, rules, law

## Abstract

The RCGP conference, like other annual healthcare conferences offers a protected space for reflection on ethical aspects of practice. This paper presents a summary and discussion of a fringe session led by the RCGP Committee On Medical Ethics at the 2017 RCGP annual conference in Liverpool. Well thought-out rules offer a potential solution to the burden of responsibility for making every single decision from first principles. But guidelines can be difficult to follow, too numerous to know, may conflict with each-other and may not be appropriate in all circumstances. Delegates at this meeting discussed barriers to good guideline development and implementation, perceptions of medicolegal risk in non-adherence, aspects of benefit, harm and justice in guideline use and ethical guidelines. Delegates found it easier in the meeting to critique clinical rather than ethical guidelines. There was broad agreement that understanding how to practice in relation to guidelines represented a learning need in general practice education.

**Why this matters to us**

The RCGP conference, like other annual healthcare conferences offers a protected space for reflection on ethical aspects of practice. In a healthcare environment that can be perceived as litigious and adversarial, guidelines offer a course of action when the challenges are unfamiliar, and possible immunity from criticism because they represent professional consensus. But guidelines can be difficult to follow, too numerous to know, may conflict with each-other and may not be appropriate in all circumstances. The RCGP committee on medical ethics accordingly chose this as the theme for an informal conversation with delegates in one of the fringe sessions of the annual conference.

**Key message**

Clinicians and policymakers alike need to be mindful that both the development and the implementation of guidelines for healthcare can be complex, difficult and even ethically-problematic. A critical approach to both clinical and specifically ethical guidelines includes a consideration of when to follow and when not to follow a guideline.

## Background

The following article presents a summary and discussion of a fringe session led by the RCGP Committee On Medical Ethics (COME) at the 2017 RCGP annual conference in Liverpool (RCGPAC). The following members of the RCGP COME were present: Simon Gregory, Andrew Papanikitas, Carey Lunan, Paul Myres, John Spicer, Alex Lee, and Mark Free. In the group of approximately 50 delegates, there was a mixture of GP Trainers, GPs with roles in adult and child safeguarding, GPs with roles in clinical guideline development, GP commissioners, Academic GPs and GP trainees. Not everyone disclosed their roles and many held several so it is not meaningful to discuss the proportion of each save that there was a definite trainee presence making up at least one tenth of the audience. Voices were present from the standing group on overdiagnosis and the ‘Choosing wisely’ campaign. The meeting was conducted under Chatham House rule, which meant that whilst all present agreed to a document summarising and discussing the ideas should be publically available, comments and ideas below are not attributable to any one individual present at the meeting. Importantly the following discussion does not represent the opinion of the RCGP or the COME or any other group represented at the meeting. The meeting followed a similar format to a session on survival and flourishing at the 2016 RCGPAC and a session on the form of RCGP ethics support and education at the 2015 RCGPAC [1]. An LJPC Paper on benefits and harms in medicine with relevance to this discussion [2] was circulated to conference delegates via Twitter (#RCGPAC) by way of some background reading –and readers of the paper attending RCGPAC were also invited to attend the fringe session. In this paper, Margaret McCartney argues that many guidelines make recommendations that are not supported by good evidence, and that even those that are should be interpreted in the context of the consultation. Thus she argues that clinicians have a general duty to be critical in the application of evidence-based medicine. The theme will be further explored at a meeting to be held on March 7th at the Royal Society of Medicine (see below).

## Introduction: the guideline spectrum

A guideline is a type of rule to guide action. Implicit in the term ‘guideline’ is the idea that it should be followed if appropriate but that it need not be if not. At the outset of the discussion we discussed why rules in general are desirable, and why we follow them. Well thought-out rules offer a potential solution to the burden of responsibility for making every single decision from first principles. There is a spectrum to the binding nature of the rules that healthcare professionals follow from statute and case law, to ethical and clinical guidelines, to general and individual/institutional policy, to guidelines representing consensus on good practice, to incentivised practices, to “rules of thumb”. The audience were keen to distinguish guidelines from protocols -a protocol is an agreed action to be followed in predefined circumstances.

People follow guidelines for numerous reasons. They may believe that guidelines represent the answer to the question, ‘What should I do?’ This is because they believe in the evidence or endorse the values (and it is important to recognise the distinction between fact and value) of the rules and possibly of the rule-makers. There are elements of faith and even religiosity in following clinical guidelines [3].

They also follow guidelines because of perceived penalty in deviation from (to avoid getting into trouble) and reward for adherence to (implicit in incentivised medicine such as the QOF) a guideline. People may also follow guidelines out of habit and the expectation that we should do so. Guidelines can add a comfortable predictability to life. Guidelines may also offer the chance to avoid making or taking responsibility for a difficult decision [4].

## Clinical guidelines and medicolegal risk

In Western medicine, clinicians have adopted a professional duty to practice evidence-based medicine. Rightly or wrongly, guidelines represent a trusted source of this evidence. The implication that follows is that deviation from clinical guidelines means practicing non-evidenced-based medicine thus falling short of an accepted standard of care. The group were concerned about what would happen to practitioners who did not follow the guidelines –were they at medicolegal risk? Debate followed as to whether clinical guidelines are statements of intent, a way of disseminating medical updates, or a responsible consensus on good practice and therefore standards of care.

Trainees at the meeting were particularly eager to discuss when and how one should not use guidelines, expressing anxiety that not following evidence-based guidelines could be seen as practicing badly. There was broad assent in the room that knowing when and when not to follow one was a key educational need for GPs. They acknowledged that awareness of a guideline was important, as was the need to explicitly record any decision not to follow one. An example given was where a guideline was not applicable to individual patient circumstances. A greater focus on individual interpretation and shared decision-making was called for, whether embedded in or in the implementation of clinical guidelines. Clinical guidelines, it was argued, could however still be compatible with movements such as ‘Realistic Medicine’ [5] Realistic Medicine puts the person receiving health and care at the centre of decision-making and encourages a personalised approach to their care. Its aims of reducing harm and waste, tackling unwarranted variation in care, managing clinical risk, and innovating to improve, are essential to a well-functioning and sustainable NHS.

## Barriers to good guideline use

The group reflected on other relevant sessions at the conference, including one presentation that suggested that the proliferation of guidelines meant that it was no longer possible to know them all, much less to critique them. The group reflected on whether having inadequate time to read every guideline was professionally acceptable. It was suggested that the defence of ‘Too busy to read the guideline’ has never been used in a UK court of law in negligence lawsuits. It was suggested that perhaps there was a need for guideline on how to use guidelines! Those present at the discussion who were involved in drafting guidelines responded that this is the rationale for producing relevant summaries such as the NICE Clinical Knowledge Summaries. Comment was made about the variety of ‘Update courses’ for GPs that work to summarise changes in guidance and clinical evidence for busy clinicians. They were also asked to what degree the wider system is taken into account during guideline compilation. One delegate present had been involved in working on writing guidelines in collaboration with secondary care colleagues, describing how the challenges around healthcare delivery differed between primary and secondary care settings. The aim was to produce guidance and standards that were relevant to the primary care setting and not just to specialists.

The group also discussed the potential for poor clinical practice because a test or treatment was financially incentivised, having been mandated by a guideline. Discussion followed about whether incentives had the potential to improve clinical practice or whether this was an inherently ethically flawed approach. We were reminded that any kind of payment for work can be ‘gamed’ unless there are ethical safeguards in place [6].

## Benefits harms and justice in guidelines use

Guidelines may have a role in managing resources appropriately. This echoes the broader ethical approach of maximising welfare via rules rather than maximising welfare for each individual case. Examples of this that were discussed included referral guidelines and prescribing guidelines. There was debate about whether guidelines made either referral from primary to secondary care or prescription more rather than less likely, and whether or not this was a good or bad thing. We discussed the use of guidelines in prescribing; ‘switching’ patients from more expensive to cheaper or more cost effective medications, restricting prescriptions for medicines that might be available to buy cheaply or which were deemed too expensive. Offering a blanket approach to prescribers offers a potential route to fairness. This also linked to the idea that ethical judgements should be made by society based on rationality and not at the bedside [7]. Moreover guidelines avoid inconsistent advice when patients present to multiple clinicians across different parts of the healthcare system.

However, guidelines as a route to fair and evidence-based healthcare was critiqued in the group. One critique linked this to the phenomenon of over-diagnosis [2,8]. Guideline cynicism was explored in a discussion of the involvement of external interests in guidelines development (such as lobby groups of the pharmaceutical industry) as well the possibility that some screening test or interventions may not confer benefit in terms of survival. In other words – whilst such interventions perform well in detecting or changing (for example) some kind of biomarker, this does not translate into a meaningful improvement in length or quality of life for the patient. The group also discussed the nature and purpose of guideline-based medicine being aimed at improving population health and saving money in health care systems, rather than benefitting individuals. For some it was suggested that following a guideline might be positively harmful with the challenge of caring for frail elderly and the inappropriateness of single-disease guidelines was cited. Competing priorities in a guideline-driven the consultation was also a source of possible harm because of competing agendas: for example the slightly far-fetched scenario of a patient presenting with domestic violence to a clinician who wished instead to prioritise managing their high blood pressure.

We discussed the importance of clinical judgement and the application of ethical principles to produce good guidelines -or good practice consistent with good guidelines. The potential use of the ‘four principles and scope’ approach as “discovery tools” to unpack the issues within consultations was discussed. Principles are not guidelines – it is not the principles that need to be balanced but the issues that they expose [9].

## Ethical guidelines, risk and doctors’ educational needs

There is “agency” (ability to make decisions) in the interpreting of guidelines – a clinician can choose to follow or not follow a guideline and may be called upon to justify either course of action. Indeed it was suggested in the meeting that such agency was an element of medical professionalism. It was argued that anyone can follow a guideline, but healthcare professionals (including GPs) have to interpret and skilfully apply guidelines based on their clinical judgement (which can include considering the patient’s wishes and narrative as well as other non-biomedical factors) However, the importance of involving patients in decision-making and in documentation of reasons not to follow a guideline were repeated many times in the discussion. The group suggested that there is even a duty to override guidelines if they are clearly inappropriate.

The group made explicit reference to the ‘Montgomery’ case whilst discussing guidelines for consent and what is required for informed consent. In this case a pregnant woman was not given full information about the risks of normal delivery against those of caesarean section despite having many risk factors for obstructed labour. The labour obstructed resulting in severe and lasting harm to mother and child [10]. As the patient would have not chosen a normal vaginal delivery had she been fully informed, the obstetrician was held to be at fault. This provoked a discussion of whether full autonomy ever truly exists for patients due to their inherent lack of medical knowledge. Paradoxically this would require a level of expertise that would negate seeking a medical opinion in the first place. The group also reflected on the availability of the internet for patients to rapidly consult guidelines themselves and use them in questioning any clinical decision. In the negotiation between different points of view, guidelines can be called upon in support of a particular course of action.

Guidelines offer a more consistent approach to patient queries and clinical presentations especially when reconciling differences of opinion. An example was given of the case where the patient desires the combined oral contraceptive pill but has risk factors that would normally preclude its use. The conflict of respecting the patient’s autonomy with avoiding harm (non-maleficence) is ultimately reconciled by the clinician. This case was one of several in a special issue of InnovAIT distributed to conference delegates [11]. Research in the context of referral guidelines, suggests that GPs subvert referral guidelines (in their patients’ interests) which are perceived as restrictive, even if they accept the underlying reasons for the referral criteria in these guidelines [12].

## Ethical guidelines

In this Fringe session there was a strong distinction made between clinical guidelines (about correct medical treatment) and ethical guidelines (about good behaviour). The latter were perceived as much more difficult to engage with. One delegate described personal experience of the perceived unhelpfulness of the General Medical Council confidentiality guidelines due to lack of flexibility and pragmatism. The situation related to an underperforming care home that required data-sharing arrangements to ensure safe and high quality care, in a situation where adult safeguarding was at issue. The local general practitioners had been reluctant and slow to override confidentiality in the interests of wider patient safety and the delegate had found it hard to obtain authoritative advice from the GMC. The group suggested that confidentiality issues / queries are the most common query to the ethics advice service provided by the GMC and BMA. We reminded ourselves of the specific circumstances when confidentiality can justifiably be waived (1) with patient consent (2) to prevent significant harm to the patient or others (3) where legally mandated. The RCGP COME triggered significant debate in 2010 with their paper, ‘*Is confidentiality a con?’* which suggested that confidentiality duties for doctors are not straightforward [13–15].

We were surprised by the fear and even anger expressed by trainees in the discussion about ethical guidelines. One trainee asked, “*Is it appropriate that GMC should sit in the role of rule*-*setter, judge and executioner?*” Trainees and trainers alike at the meeting were concerned about the perceived pseudo-legal nature of GMC guidelines. To not follow GMC guidance risks sanctions. It was suggested that this may mean that they are followed uncritically even to the detriment of patient care. Conversely the group recognised the perceived obligation to follow medical defence organisations’ advice because they are providing insurance cover, and to not follow their advice may invalidate this. This led to the question of, *‘What are the training needs for future GPs in this area?’* Students and trainees are not always encouraged to think critically – a delegate gave the example that medical students do not necessarily interpret, but regurgitate GMC duties verbatim.

## Some possible conclusions

Discussion at the fringe event tapped into group perceptions of moral distress in attempting to deliver care where duties can feel impossible to meet: duty to do your best by the patient, to the wider community and to justify the resources to deliver the care. The session’s chair observed that more delegates seemed comfortable critiquing clinical guidelines than GMC guidelines. This may be because there is acceptance that one cannot know every clinical guideline, but may represent a fear that ethical guidelines are either knowable, or can be worked out from first principles and therefore there is no excuse to “do the wrong thing”. Paradoxically, the group found it easier to critique ‘medicine’ rather than ‘morality,’ suggesting that it is easier to find fault with a clinical guideline than an ethical one. In both cases ignorance is seemingly no excuse for deviating from acceptable practice.

The group recognised that guidelines can conflict and can require further interpretation, possibly generating as well as solving ethical dilemmas. The presence of a large proportion of the COME at the event, who contributed to the discussion, was helpful in keeping discussion relevant and collegial and in ensuring that conference delegates’ perspectives were heard first-hand. The COME members invited delegates to contact them (in person at the conference and in writing thereafter) with queries and ideas, and offered to publish a summary of the discussion in an accessible forum that might allow discussion to continue.

Please continue this conversation on Twitter using following hashtag and twitter handles: #RCGPAC @Thomas7Paul @LJPCjournal @gentlemedic @careylunan @johnspicer3

## Continue this conversation with members of the RCGP COME and other expert speakers at the Royal Society of Medicine and other expert speakers on 7th March 2018 at the 8th Primary Care Ethics Conference: are guidelines the answer to good medical practice?

*About the 7th March 2018 event:* In today’s practice not following a guideline can seem like acting against a responsible body of medical opinion. At this meeting delegates are invited to join an expert panel of speakers to discuss the ethics of practicing with guidelines and how best to use guidelines for ethical practice. We will discuss whether there are times where a guideline should be followed and when it should not. Is it moral or immoral to incentivise guidelines? We will discuss where guidelines come from in terms of evidence, values and politics, and whether awareness of this matters. Our panel is drawn from Academia, Education and Practice across the UK. More broadly the meeting will continue to serve as the inter-professional forum for primary care ethics with an open call for posters in this field.

For more details on registration or to submit an abstract visit

https://www.rsm.ac.uk/events/gpk04

## Governance

Members of the RCGP Committee on Medical Ethics oversaw the discussion, which was conducted as a fringe session of the RCGP Annual Conference 2017.

## Evidence of permissions to publish other people’s work

Not applicable.

## Disclosure statement

No potential conflict of interest was reported by the authors.

## Funding

This work was supported by NIHR.

## Related LJPC Papers

McCartney M (2017): Benefits, harms and evidence (online publication) – reflections from UK primary healthcare, London Journal of Primary Care, DOI: 10.1080/17571472.2017.1384610

Papanikitas A (2016): Education and debate: a manifesto for ethics and values at annual healthcare conferences, London Journal of Primary Care, 8(6): 96–99. doi: 10.1080/17571472.2016.1244152
